# Dysmenorrhea catastrophizing and functional impairment in female pelvic pain

**DOI:** 10.3389/fpain.2022.1053026

**Published:** 2023-01-06

**Authors:** Rui Li, Donna A. Kreher, Ashley L. Gubbels, Tonya M. Palermo, Amy R. Benjamin, Carrie S. Irvine, Andrea Hart, Todd A. Jusko, Christopher L. Seplaki

**Affiliations:** ^1^ Center for Child Health, Behavior & Development, Seattle Children's Research Institute, Seattle, WA, United States; ^2^ Department of Psychiatry, University of Rochester School of Medicine & Dentistry, Rochester, NY, United States; ^3^ Department of Obstetrics and Gynecology, Cleveland Clinic Foundation, Cleveland, OH, United States; ^4^ Department of Anesthesiology & Pain Medicine, University of Washington School of Medicine, Seattle, WA, United States; ^5^ Department of Obstetrics and Gynecology, University of Rochester School of Medicine & Dentistry, Rochester, NY, United States; ^6^ Department of Public Health Sciences, University of Rochester School of Medicine & Dentistry, Rochester, NY, United States

**Keywords:** dysmenorrhea, menstrual pain, chronic pelvic pain (CPP), catastrophizing, pain intensity, pain interference

## Abstract

**Background:**

Dysmenorrhea is suggested to increase the risk of chronic pain by enhancing central sensitization. However, little is known about whether emotional and cognitive responses induced by dysmenorrhea contribute to chronic pain interference. This study examined the association between catastrophizing specific to dysmenorrhea and both dysmenorrhea and chronic pelvic pain (CPP)-associated pain interference.

**Methods:**

Women (*N* = 104) receiving care for CPP through a tertiary gynecological pain clinic between 2017 and 2020 were recruited. They completed the Pain Catastrophizing Scale, the Brief Pain Inventory–pain interference, and a separate questionnaire regarding dysmenorrhea symptoms and treatment preceding the development of CPP. Dysmenorrhea catastrophizing and interference measures were developed and tested for internal consistency and construct validity. Multiple linear regression models examined dysmenorrhea catastrophizing in association with dysmenorrhea interference and CPP-associated pain interference.

**Results:**

Dysmenorrhea catastrophizing and interference measures demonstrated excellent internal consistency (Cronbach's Alpha = 0.93 and 0.92 respectively) and evidence of construct validity (correlated with dysmenorrhea severity and treatment, Ps < 0.01). Dysmenorrhea catastrophizing was moderately correlated with pain catastrophizing (*ρ* = 0.30, *P* = 0.003), and was associated with greater dysmenorrhea interference (*P* < 0.001) and CPP-associated pain interference (*P* = 0.032) accounting for general pain catastrophizing and other outcome-specific confounders. Dysmenorrhea intensity was most predictive of dysmenorrhea catastrophizing.

**Conclusion:**

Among our clinical sample of women with CPP, dysmenorrhea catastrophizing was associated with greater dysmenorrhea interference and subsequent CPP-associated pain interference. More research is needed to determine whether reduction in dysmenorrhea catastrophizing leads to reduced pain interference associated with female pelvic pain.

## 1. Introduction

Dysmenorrhea, or painful menstrual cramps, is the most common gynecological condition during women's reproductive years and can be associated with substantial interference with daily life. Over one in five young women report school absences and over 40% have reduced academic performance due to dysmenorrhea (1), with approximately 600 million lost working hours and $2 billion health cost attributed to dysmenorrhea each year in the U.S. (2). Dysmenorrhea may also predispose women to future chronic pain conditions (3, 4), including chronic pelvic pain (CPP).

Female CPP is pain perceived to be related to the pelvic organs or structures lasting more than 6 months (5, 6), and is prevalent in 14%–24% of reproductive-aged women (7–10). It often involves multiple chronic pain syndromes such as endometriosis and irritable bowel syndrome, and is associated with significant psychosocial and emotional sequelae (11). Dysmenorrhea is suggested to increase the risk of CPP given its potential role in facilitating central sensitization (12–14). However, knowledge in the behavioral mechanisms involved in the transition from dysmenorrhea to CPP is more limited, although the identification of such mechanisms is important for non-pharmacological prevention interventions.

Pain catastrophizing is one such mechanism, characterized by the tendency to magnify the threat value of a pain stimulus, to feel helpless in the context of pain, and inability to inhibit pain-related thoughts (15, 16). According to the Fear-Avoidance model of pain (17), pain catastrophizing, in the context of actual or anticipated pain experiences, causes pain-related fear which further leads to pain-related disability. Clinically, pain catastrophizing has been associated with worse post-surgical (18, 19) and chronic pain-related outcomes (20, 21), including greater severity and worse prognosis of female CPP (22, 23). Meanwhile, studies have associated pain catastrophizing with more severe dysmenorrhea, higher disability due to dysmenorrhea, and lower perceived efficacy of Over-the-Counter (OTC) medications for dysmenorrhea symptom management (24–26). A brief mind-body intervention has demonstrated reduction in menstrual pain intensity and pain catastrophizing but not in depression, anxiety, or somatization, suggesting pain catastrophizing may be a particularly important cognitive-affective response associated with dysmenorrhea (27).

Despite the relevance of pain catastrophizing in both dysmenorrhea and CPP, whether dysmenorrhea contributes to the onset and worsening of CPP *via* the mechanism of pain catastrophizing is unknown. As a recurrent and more predictable pelvic pain condition, dysmenorrhea may be associated with pain anticipation, potentially facilitating an anxious, ruminative catastrophizing process that not only increases disability due to dysmenorrhea, but over time reinforces negative cognitive-affective responses to pain and results in greater CPP-related disability. Given the prevalence and loss of productivity associated with dysmenorrhea, it is critical to gain an understanding of how symptom-related catastrophizing may contribute to interference due to dysmenorrhea. Moreover, the process of transitioning from dysmenorrhea to more complex and unpredictable CPP usually takes a decade or so (28). This provides a unique opportunity for testing whether the negative cognitive-affective responses to recurrent dysmenorrhea reinforce a maladaptive coping framework that could potentially be associated with more pain interference related to future CPP.

Thus, this study aimed to fill the gap in knowledge of the role of dysmenorrhea-specific catastrophizing in pelvic pain interference in a clinical sample of women with CPP, with the overarching hypothesis that dysmenorrhea catastrophizing would be associated with both dysmenorrhea interference and subsequent CPP interference. Given the lack of measures assessing catastrophizing specific to dysmenorrhea, we started by validating a newly developed measure of dysmenorrhea catastrophizing. We characterized dysmenorrhea catastrophizing as negative cognitive-affective responses induced by the experience of menstrual pain, that involves anxious anticipation of menstrual pain, repetitive negative thoughts about ongoing menstrual pain, overemphasis on the probability of a catastrophic outcome associated with menstrual pain, and a feeling of helplessness in the context of menstrual pain. It shares conceptual similarity with the general pain catastrophizing but is more dependent on the recurrent menstrual pain experiences. We then tested the hypothesis that dysmenorrhea catastrophizing would be associated with greater dysmenorrhea interference as well as greater subsequent CPP interference. Finally, as the Fear-Avoidance model implies that pain catastrophizing emerges in the context of pain, for exploratory analyses, we further examined the associations between a series of dysmenorrhea features (e.g., intensity, frequency) and dysmenorrhea catastrophizing, with the goal to inform future studies of dysmenorrhea catastrophizing.

## 2. Materials and methods

### 2.1. Setting and study population

The Center for Chronic Pelvic and Vulvar Pain (Center) at the University of Rochester Medical Center (URMC) is a tertiary gynecological referral center for chronic pelvic and/or vulvar pain in Rochester, New York, USA. Patients are referred from a wide catchment area in upstate New York. The Center is staffed by gynecologists with expertise in management of chronic pelvic/vulvar pain, a clinical psychologist specializing in behavioral treatment of chronic pain, a pelvic health physical therapist, clinical fellows, and registered nurses. Beginning in October 2017, new patients referred to the Center received a standardized set of questionnaires prior to their first appointment to collect information regarding their pelvic pain symptoms (including pain intensity and interference), medical history, demographic and psychosocial measures (including pain catastrophizing). These questionnaires were administered as part of standard intake for new patient evaluation.

Our study population comprised women ages ≥18 years who had non-cyclic pelvic and/or vulvar pain for at least 6 months and were referred to our Center for evaluation and treatment for their pain. Exclusion criteria were (1) age under 18 years; (2) pregnancy at the clinical visit; (3) treatment for cancer-associated pain; (4) incomplete standardized intake questionnaires; (5) cognitive impairment as judged by the researcher; and (6) non-English speaking.

### 2.2. Study design

This is a cross-sectional study of women with chronic pelvic and/or vulvar pain who received treatment at the Center between October 2017 and March 2020. Pelvic/vulvar pain intensity and interference, demographic, and psychosocial data were obtained from the standardized intake questionnaires (administered as part of standard clinical care). Dysmenorrhea frequency, duration, and intensity, dysmenorrhea catastrophizing, dysmenorrhea interference, and dysmenorrhea treatment history prior to the development of chronic pelvic/vulvar pain were obtained from a patient-completed dysmenorrhea questionnaire that was specifically developed for this study. The Research Ethics Board of the University of Rochester Medical Center, Rochester, NY, USA approved this study (STUDY00004434, MOD00005488).

### 2.3. Recruitment

Recruitment took place between January and August 2020. Between January and Mid-March, eligible patients were approached in-person in the clinic, either before or after they finished the evaluation with a gynecologist. They were provided detailed information regarding the purpose and procedure of the study and provided a written informed consent if they agreed to participate. Women who agreed to participate then completed the paper-based dysmenorrhea questionnaire (described above) on site which took about 5 min to complete.

Due to the COVID-19 pandemic, recruitment for the remainder of the study was completed online from March-August 2020. Eligible patients who had previously agreed to be contacted for future research opportunities were contacted by phone. The study was briefly explained, and those who expressed interest in participating were given the options of completing the dysmenorrhea questionnaire over the phone, or online *via* REDCap electronic data capture tools hosted at the University of Rochester Medical Center (29, 30).

### 2.4. Measures

A dysmenorrhea questionnaire was developed to measure dysmenorrhea symptoms and treatments prior to the development of chronic pelvic/vulvar pain for which women were referred to the Center (see Supplementary Material for the questionnaire). The questionnaire measured the onset, frequency, duration, intensity, catastrophizing, interference, and treatment history of dysmenorrhea. The questionnaire was designed based on expert opinions and has gone through multiple internal iterations within the research team consisting of gynecologists, a clinical psychologist, pain researchers, and an epidemiologist, and was further pilot-tested among a group of female graduate students both with and without dysmenorrhea. In order to help participants better differentiate between dysmenorrhea and CPP, simple definitions were provided at the beginning of the questionnaire: “*Period cramps are crampy pains you get just before or during your period*”, “*Chronic pelvic pain is pain in the pelvis or vulva that is not related to your menstrual cycle, although you may still have period cramps.*” Participants were repeatedly reminded throughout the questionnaire that they should answer based on their experience prior to the onset of their CPP.

#### 2.4.1. Dysmenorrhea catastrophizing

Dysmenorrhea-specific catastrophizing measure was adapted from the Pain Catastrophizing Scale (PCS) (16), the most widely used measure for pain catastrophizing across different populations and which has good internal consistency, construct validity, and test-retest reliability (15, 31). Because the PCS measures responses to pain in general, which may be inadequate in capturing context or disease-specific catastrophizing, particularly in regard to previous dysmenorrhea symptoms, we constructed a dysmenorrhea catastrophizing measure by adopting three items from the PCS that explain the highest variance of each of the 3 dimensions of pain catastrophizing—rumination, magnification, and helplessness (16). Participants were asked to recall the period prior to developing their chronic pelvic and/or vulvar pain. They responded to how much the following statements applied to them when they were experiencing period cramps: “*I kept thinking about how much it hurt*” for dysmenorrhea rumination, “*I was afraid that the pain would get worse*” for dysmenorrhea magnification, and “*It was awful, and I felt that it overwhelmed me*” for dysmenorrhea helplessness, respectively. Given the cyclic nature of dysmenorrhea, we added a fourth item asking the degree to which participants felt nervous **before** their menstrual period, to capture anxious anticipation of menstrual pain (i.e., “*Before my period, I became nervous about my period cramps*”). For each item, answer choices included “Not at all”, “To a slight degree”, “To a moderate degree”, and “To a great degree”, coded 1–4. In determining the effect of dysmenorrhea catastrophizing on dysmenorrhea interference and CPP interference, dysmenorrhea catastrophizing was the independent variable, calculated as the mean score (ranged between 1 and 4) based on answers to the 4 items from the dysmenorrhea catastrophizing measure. A higher score indicated a higher level of dysmenorrhea catastrophizing.

#### 2.4.2. Dysmenorrhea interference

Dysmenorrhea interference was measured by three items adapted from the Brief Pain Inventory-Pain Interference (BPI-PI) (32). BPI-PI is a widely used tool for assessing the impact of pain on functioning (32). We constructed our dysmenorrhea interference measure using three items adapted from the BPI-PI that maintain its two major subdomains—activity and social functioning. Items assess the interference of dysmenorrhea with participants' general daily activity (e.g., eating, bathing, dressing, walking), school/work activity (e.g., attending classes, taking exams, performing normal work), and relations with others (e.g., going out with friends, physical intimacy). Similarly, participants were asked to recall the period prior to developing their chronic pelvic and/or vulvar pain. Answer choices for each item included “Did not interfere at all”, “Interfered a little”, “Interfered a lot”, and “Completely interfered”, coded 1–4. The degree of dysmenorrhea interference was calculated as the mean score (ranged between 1 and 4) based on answers to the 3 items from the dysmenorrhea interference measure, with a higher score indicating a higher level of dysmenorrhea interference.

#### 2.4.3. CPP-associated pain interference

Current pain interference was measured using the BPI-PI (32) which asked the patient's pain interference with general activity, mood, walking ability, normal work, relationship with other people, sleep, and enjoyment of life, during the past month. A mean score based on the seven items was calculated, with a higher score indicating a higher level of pain interference. As the BPI-PI measures general pain interference, and many participants may have chronic non-pelvic pain, we used the BPI-PI mean score to indicate CPP-associated pain interference for which CPP may or may not be the main contributor.

#### 2.4.4. Other clinical information

Participants' sociodemographic and psychosocial information was collected from the intake questionnaires or the electronic health record (EHR). Age (years), race and ethnicity (classified as non-Hispanic white, non-Hispanic black, Hispanic, and others; merged into non-Hispanic white and others for multivariable analyses due to the small sample size), body mass index (BMI: kg/m^2^), and use of tobacco (yes vs. no) were extracted from the EHR. Education attainment (high school, associate's degree, college or bachelor's degree, and master's or doctoral degree, coded 1–4) and abuse history (yes vs. no, both in childhood and adulthood) were obtained from the intake questionnaire. Self-report of any sexual, physical, or emotional abuse as a child or teenager was classified as experience of childhood abuse. Self-report of any sexual, physical, or emotional abuse as an adult was classified as experience of adulthood abuse.

General pain catastrophizing at the initial clinical visit was measured by the PCS total score, with a higher score indicating a higher level of pain catastrophizing (16). Dysmenorrhea intensity was indicated by the overall intensity of menstrual cramps without medication measured by a Likert scale with responses including none, mild, moderate, severe, worst pain imaginable (coded 0–4). CPP intensity was indicated by the average level of CPP in the past month measured at the initial clinical visit using a Likert scale (0–4 for none, mild, moderate, severe, worst possible). Current or previous diagnoses of common chronic pain syndromes associated with female CPP were collected as a combination of medical record extraction, physical exam, and patients' self-report. Patients also completed screening of depression and anxiety at the initial clinical visit using the Patient Health Questionnaire-2 (PHQ-2) (33) and Generalized Anxiety Disorder-2 (GAD-2) (34) and those scoring more than 3 for either received clinical assessment of major depressive disorder (MDD) and generalized anxiety disorder (GAD).

### 2.5. Statistical analyses

#### 2.5.1. Validation of dysmenorrhea catastrophizing and interference measures

We evaluated measurement properties of the 4-item dysmenorrhea catastrophizing and 3-item dysmenorrhea interference measures as follows. We assessed internal consistency by calculating the Cronbach's Alpha. To assess construct validity, we examined Spearman correlation coefficients between dysmenorrhea frequency (1–4, lowest to highest), dysmenorrhea duration within a typical menstrual cycle (1–3, lowest to highest), dysmenorrhea intensity (1–4, lowest to highest), and dysmenorrhea catastrophizing and dysmenorrhea interference, respectively. We expected moderate to high correlations between dysmenorrhea severity and dysmenorrhea catastrophizing and interference. Further for construct validity, we compared dysmenorrhea catastrophizing and dysmenorrhea interference scores by ever seeking health care specifically for dysmenorrhea (yes vs. no), ever being prescribed opioid pain medications for managing dysmenorrhea (yes vs. no), and ever using marijuana for managing dysmenorrhea (yes vs. no), assuming higher dysmenorrhea catastrophizing and interference would correspond to more health care seeking and the use of opioids and marijuana. Additionally, we summed the number of self-reported management strategies for dysmenorrhea and calculated the Spearman correlation coefficients with dysmenorrhea catastrophizing and dysmenorrhea interference, assuming moderate to high correlations. To evaluate discriminant validity, we hypothesized that the PCS total score would have higher correlation with dysmenorrhea catastrophizing than dysmenorrhea interference, and that the BPI-PI mean score would have higher correlation with dysmenorrhea interference than dysmenorrhea catastrophizing.

#### 2.5.2. Association between dysmenorrhea catastrophizing and dysmenorrhea interference

We fit multiple linear regression models for the association between dysmenorrhea catastrophizing and dysmenorrhea interference, adjusting for pre-defined confounders selected based on their potential associations with both dysmenorrhea catastrophizing and dysmenorrhea interference without being on the causal pathway between the two. Confounders included dysmenorrhea severity measures including dysmenorrhea frequency (1–4), dysmenorrhea duration (1–3), and dysmenorrhea intensity (1–4); pain catastrophizing (PCS total score) which was conceptually associated with dysmenorrhea catastrophizing and may increase dysmenorrhea interference; demographic variables including age (years), education attainment (1–4), and race and ethnicity (non-Hispanic white vs. other racial and ethnic groups combined); diagnosis of endometriosis (the most common cause of secondary dysmenorrhea); and experience of childhood abuse (yes vs. no) which has been associated with dysmenorrhea (35) and pain catastrophizing (36). To assess the possibility that any association between dysmenorrhea catastrophizing and dysmenorrhea interference is due to pain catastrophizing, we repeated the above models without including dysmenorrhea catastrophizing as a predictor to isolate the effect of pain catastrophizing on dysmenorrhea interference.

#### 2.5.3. Association between dysmenorrhea catastrophizing and CPP-associated pain interference

We fit multiple linear regression models for the association between dysmenorrhea catastrophizing and CPP-associated pain interference (BPI-PI mean score), adjusting for pre-defined confounders selected based on their potential associations with both dysmenorrhea catastrophizing and CPP interference without being on the causal pathway between the two. Confounders included pain catastrophizing, demographic variables (age, education attainment, and race and ethnicity), and experience of childhood abuse. We additionally adjusted for CPP intensity (0–4), diagnosis of MDD (yes vs. no), diagnosis of GAD (yes vs. no), and experience of adult abuse (yes vs. no). These variables may not be directly associated with dysmenorrhea catastrophizing but are predictive of CPP-associated pain interference; adjusting for these variables would enhance the precision of the estimate of the association between the exposure (dysmenorrhea catastrophizing) and outcome (CPP-associated pain interference). Similarly, we repeated the above models without including dysmenorrhea catastrophizing as a predictor to isolate the effect of pain catastrophizing on CPP-associated pain interference.

#### 2.5.4. Exploratory analyses: Predictors of dysmenorrhea catastrophizing

We performed simple linear regression models to examine dysmenorrhea catastrophizing in association with age, race and ethnicity, education, dysmenorrhea onset, frequency, starting time, duration, intensity, and use of opioids and marijuana. In these bivariate analyses, we calculated coefficient of determination (*R*^2^) to indicate the variance in the dependent variable, dysmenorrhea catastrophizing, that was explained by each individual predictor. Significant predictors at an alpha level of 0.05 then entered a multiple linear regression model for predicting dysmenorrhea interference. Importance of predictors were determined based on the explanatory power of individual predictors as well as the *P* values in the multivariable analysis. Data clean, management, and statistical analyses were performed in SAS v.9.4 (SAS Inc., Cary, NC, USA).

## 3. Results

### 3.1. Sample characteristics

**Figure 1:**
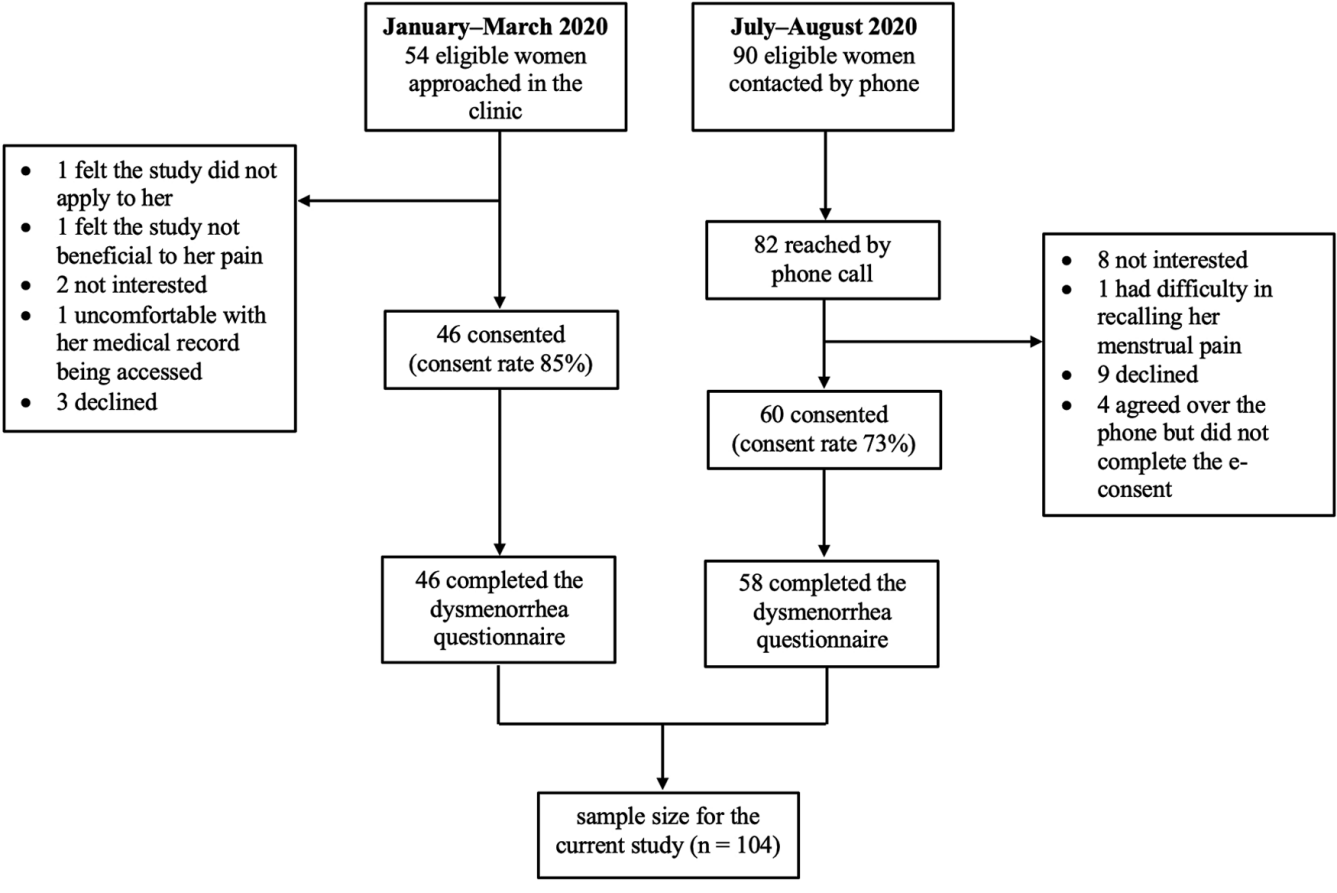
Flow chart of recruitment of the study population.

A total of 104 women were recruited, with 44% recruited in-person and 56% recruited remotely (Figure 1). The consent rate was 85% for in-person recruitment and 73% for remote recruitment. As shown in Table 1, the study sample were primarily aged between 18 and 50 years, non-Hispanic white, and with a college or higher degree. Most presented with chronic pelvic pain and 27% mainly presented with chronic vulvar pain (still classified as chronic pelvic pain under the current IASP classification system). A full description of pelvic pain onset, duration, intensity, diagnoses, pain catastrophizing and pain interference is also presented in Table 1.

**Table 1:** Characteristics of the study sample (*n* = 104).

	*n* (%) or mean (±SD) or median (25th–75th)
Age at the clinical visit (years)	35.8 (±13.6)
18–25	27 (26.0%)
26–35	36 (34.6%)
36–50	25 (24.0%)
51–70	16 (15.4%)
Race/ethnicity	
Non-Hispanic white	84 (80.8%)
Non-Hispanic black	3 (2.9%)
Hispanic	6 (5.8%)
Others	11 (10.6%)
Highest education completed	
High school or equivalent	24 (23.1%)
Associate's degree	19 (18.3%)
College/Bachelor's degree	34 (32.7%)
Master's or doctoral degree	21 (20.2%)
Unknown	6 (5.8%)
Marital status	
Single	36 (34.6%)
Married	43 (41.3%)
Partnered	17 (16.3%)
Divorced	5 (4.8%)
Widowed	3 (2.9%)
Employment	
Full-time	43 (41.3%)
Part-time	15 (14.4%)
Not working	39 (37.5%)
Unknown	7 (6.7%)
BMI at initial visit (kg/m^2^)	28.0 (±8.4)
<18.5	4 (3.8%)
18.5–24.9	35 (33.7%)
25.0–29.9	36 (34.6%)
≥30	29 (27.9%)
Ever used tobacco	34 (32.7%)
Childhood sexual abuse	22 (21.1%)
Childhood physical or emotional abuse	23 (22.1%)
Any childhood abuse	37 (35.6%)
Adult sexual abuse	24 (23.1%)
Adult physical or emotional abuse	35 (33.7%)
Any adult abuse	44 (42.3%)
Age of non-cyclical pelvic/vulvar pain onset: range = 10–70	27.2 (±13.9)
Years with chronic pelvic/vulvar pain: range = 0–43	6.3 (2–11.5)
Pelvic/vulvar pain intensity: Likert scale 0–4^a^	2.3 (±0.9)
Pain catastrophizing (PCS total score, 0–52)	26.5 (±11.9)
Pain interference (BPI-PI, 0–10)	5.2 (±2.8)
MDD	59 (56.7%)
GAD	46 (44.2%)
Endometriosis	37 (35.6%)
Bladder pain syndrome	19 (18.3%)
Irritable bowel syndrome	45 (43.4%)
Vulvodynia	38 (36.5%)
Myofascial pelvic pain	63 (60.6%)
Fibromyalgia	14 (13.5%)

SD, standard deviation; BMI, body mass index; NRS, numerical rating scale; PCS, Pain Catastrophizing Scale; BPI-PI, Brief Pain Inventory-Pain Interference; MDD, major depressive disorder; GAD, generalized anxiety disorder.

^a^ Pelvic/vulvar pain intensity was measured using the question: Please rate the following about your pelvic or vulvar pain (in a typical month): none, mild, moderate, severe, worst possible.

Table 2 summarizes dysmenorrhea symptoms and treatment history prior to the development of CPP. Overall, 55% of our sample reported severe or worst dysmenorrhea beginning within 1 year of their first menstrual period, that occurred during each or most of their menstrual periods. However, only 44% of our sample reported seeking medical treatment specifically for their dysmenorrhea symptoms. The most common management strategies were over-the-counter (OTC) pain medications, heating pad, and various hormonal treatments. Of note, 9% reported being prescribed opioid pain medications and 11% reported using marijuana for managing dysmenorrhea symptoms.

**Table 2:** Dysmenorrhea symptoms and treatment among the study sample (*n* = 104).

	*n* (%) or mean (±SD)
Had dysmenorrhea ever: yes^a^	103 (100%)
Age of first menstrual period: range = 8–17^b^	12.1 (±1.4)
Age of dysmenorrhea onset: range = 8–45^c^	13.7 (±4.7)
With 1st/2nd period	59 (57.3%)
≤1 year after 1st period	28 (27.2%)
≤5 years after 1st period	9 (8.7%)
>5 years after 1st period	7 (6.8%)
Missing^d^	1
Frequency of dysmenorrhea	
During every period	73 (70.9%)
During most of periods	15 (14.6%)
During some periods	9 (8.7%)
Seldom or rarely	6 (5.8%)
Missing	1
When dysmenorrhea usually started	
The day period started	35 (34.0%)
1–2 days before the period	49 (47.6%)
3 or more days before the period	19 (18.4%)
Missing	1
Usual duration of dysmenorrhea	
1–2 days	33 (32.0%)
3–4 days	38 (36.9%)
≥5 days	32 (31.1%)
Missing	1
Intensity of dysmenorrhea without medication	
Mild	15 (14.6%)
Moderate	26 (25.2%)
Severe	57 (55.3%)
Worst pain imaginable	5 (4.9%)
Missing	1
Linear score for intensity (1–4)	2.5 (±0.8)
Ever sought treatment specifically for dysmenorrhea	46 (44.2%)
Management history of dysmenorrhea	
OTC (Ibuprofen, Naproxen, Aspirin, Tylenol)	97 (93.3%)
Heating pad	82 (78.8%)
Hormonal treatment (any)	55 (52.9%)
Birth control pills, patch, ring	55 (52.9%)
Progesterone only pills	11 (10.6%)
Depo Provera	10 (9.6%)
Implant	4 (3.8%)
Progestin IUD	11 (10.6%)
Prescription gabapentin, muscle relaxants, or NSAIDs	18 (17.3%)
Prescription opioid pain medications	9 (8.7%)
Marijuana	11 (10.6%)
TENS	4 (3.8%)
Surgical interventions (spinal manipulation, laparoscopy)	10 (9.6%)
Complementary medicine (fish oil, acupuncture, yoga)	15 (14.4%)

SD, standard deviation; OTC, over-the-counter; IUD, intrauterine device; NSAIDs, non-steroidal anti-inflammatory drugs; TENS, transcutaneous electrical nerve ablation.

^a^ One participant did not report whether she had dysmenorrhea.

^b^ Age of first menstrual period was missing for 2 participants.

^c^ Age of dysmenorrhea onset was missing for 2 participants.

^d^ Percentages were calculated for non-missing values.

### 3.2. Measurement properties of dysmenorrhea catastrophizing and interference

**Figure 2:**
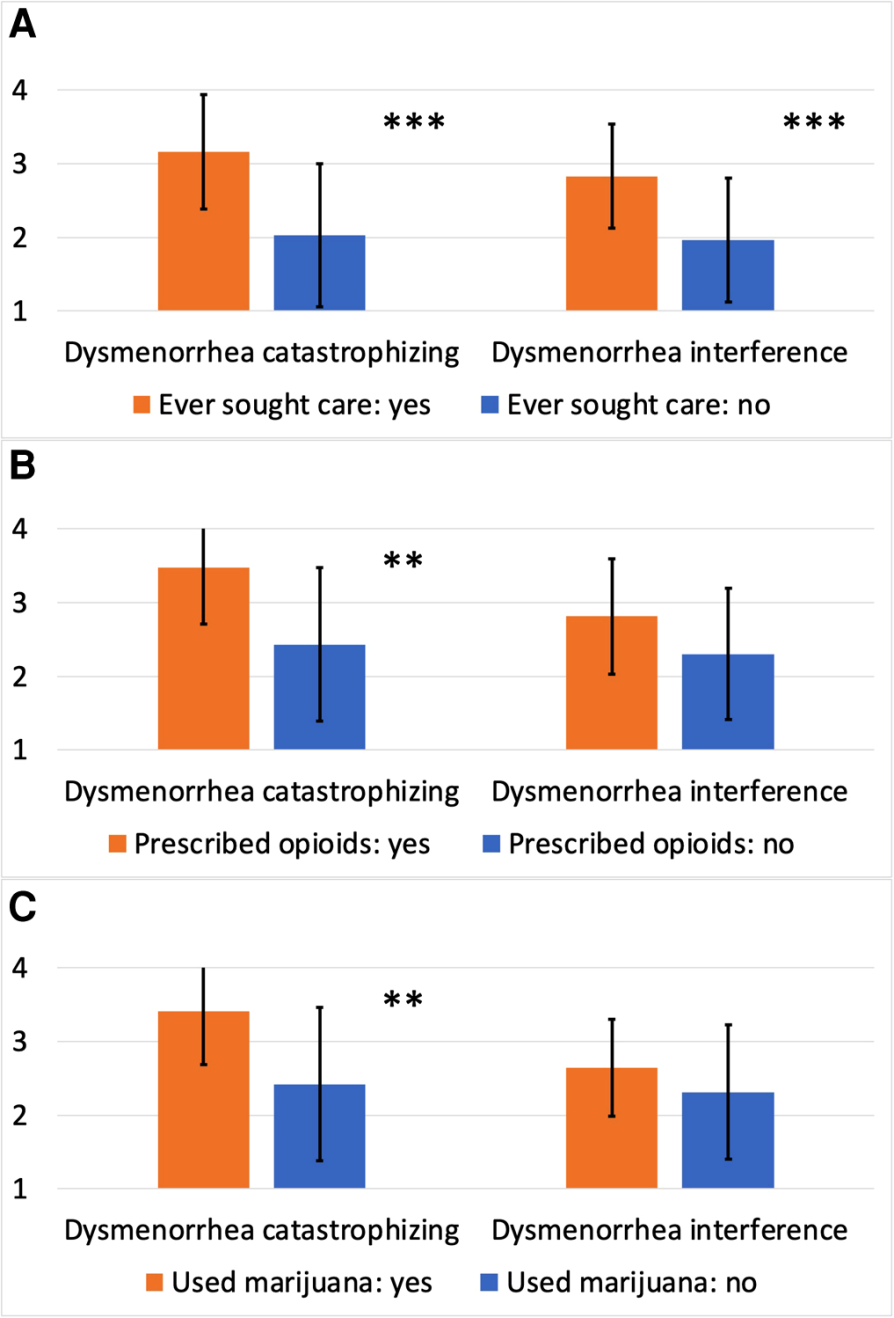
Dysmenorrhea catastrophizing and dysmenorrhea interference scores by treatment history. (**A**) Dysmenorrhea catastrophizing and dysmenorrhea interference scores by ever seeking care specifically for dysmenorrhea; (**B**) dysmenorrhea catastrophizing and dysmenorrhea interference scores by ever being prescribed opioid medications for managing dysmenorrhea; (**C**) dysmenorrhea catastrophizing and dysmenorrhea interference scores by ever using marijuana for managing dysmenorrhea. ***P < 0.001; **P < 0.01.

As shown in Table 3, dysmenorrhea catastrophizing and dysmenorrhea interference measures both had excellent internal consistency (Cronbach's Alpha = 0.93 and 0.92, respectively). The mean score for dysmenorrhea catastrophizing and dysmenorrhea interference rated on a 1–4 scale was 2.53 and 2.35, respectively. As expected, dysmenorrhea catastrophizing was strongly correlated with dysmenorrhea frequency, duration, and intensity (*ρ* between 0.56–0.73, Ps < 0.001), as well as a greater number of strategies used for managing dysmenorrhea (*P* < 0.001). Higher dysmenorrhea catastrophizing scores were seen among those seeking health care for dysmenorrhea (*P* < 0.001, Figure 2A), those being prescribed opioid pain medications for managing dysmenorrhea (*P* < 0.01, Figure 2B), and those using marijuana for managing dysmenorrhea (*P* < 0.01, Figure 2C). However, dysmenorrhea catastrophizing was only moderately correlated with the PCS total score (*ρ* = 0.30, *P* < 0.01), suggesting some independence of the measures. As expected, dysmenorrhea interference showed moderate to strong correlations with dysmenorrhea frequency, duration, and intensity (*ρ* between 0.44–0.75, Ps < 0.001), as well as a greater number of strategies used for managing dysmenorrhea (*P* < 0.001). Higher dysmenorrhea interference scores were seen among those seeking health care for dysmenorrhea (*P* < 0.001, Figure 2A), and among those being prescribed opioid medications or using marijuana (differences were not statistically significant, Figures 2B,C). The PCS total score demonstrated stronger correlation with dysmenorrhea catastrophizing compared with dysmenorrhea interference, while the BPI-PI mean score demonstrated stronger correlation with dysmenorrhea interference compared with dysmenorrhea catastrophizing.

**Table 3:** Distributions, internal consistency and construct validity of dysmenorrhea catastrophizing and dysmenorrhea interference (*n* = 100)^a^.

	Dysmenorrhea catastrophizing	Dysmenorrhea interference
Means	2.53	2.35
Standard deviation	1.06	0.89
Range	1–4	1–4
Internal consistency: Cronbach's Alpha	0.93	0.92
Correlation with dysmenorrhea frequency	0.56***	0.53***
Correlation with dysmenorrhea duration	0.58***	0.44***
Correlation with dysmenorrhea intensity	0.73***	0.75***
Correlation with dysmenorrhea interference	0.78***	-
Correlation with pain catastrophizing (PCS)	0.30**	0.26*
Correlation with pain interference (BPI-PI)	0.34***	0.42***
Correlation with the number of strategies used for managing dysmenorrhea (0–12)	0.53***	0.53***

PCS, Pain Catastrophizing Scale; BPI, Brief Pain Inventory-Pain Interference.

* *P* < 0.05.

** *P* < 0.01.

*** *P* < 0.001.

^a^ Dysmenorrhea catastrophizing and dysmenorrhea interference were missing for 4 participants.

### 3.3. Dysmenorrhea catastrophizing in association with dysmenorrhea interference and CPP-associated pain interference

Overall missingness ranged from 1.0% for dysmenorrhea intensity to 7.7% for the PCS total score. Out of the 104 participants, complete data were available for 83 (80%). Comparing those with complete data to those with any missing data, there were no differences in dysmenorrhea catastrophizing, dysmenorrhea interference, or CPP-associated pain interference; however, those with missing data were older (*P* = 0.093), had lower education attainment (*P* = 0.072) and higher pain catastrophizing (*P* = 0.024). There were no group differences in other covariates. For enhancing statistical power and reducing potential bias due to imbalanced confounders, we performed multiple imputation, which is a general approach to the problem of missing data. It allows for the uncertainty about the missing data by creating several different plausible imputed data sets and appropriately combining results obtained from each of them (37). We generated 10 imputed datasets assuming multivariate normal distribution (see Tables 4, 5 for variables used for imputation). Multiple linear regression models were fit on the imputed datasets.

**Table 4:** Multiple linear regression model for the covariates-adjusted association between dysmenorrhea catastrophizing and dysmenorrhea interference^a^.

Variables predicting dysmenorrhea interference (1–4)	Beta (95% CI)	*P*
Dysmenorrhea catastrophizing (1–4, lowest to highest)	0.44 (0.29, 0.59)	<0.001
Dysmenorrhea frequency (1–4, lowest to highest)	0.12 (−0.05, 0.29)	0.167
Dysmenorrhea duration (1–3, lowest to highest)	−0.15 (−0.34, 0.04)	0.122
Dysmenorrhea intensity (1–4, lowest to highest)	0.29 (0.07, 0.50)	0.009
Pain catastrophizing (PCS total score)	0.00 (−0.01, 0.01)	0.397
Age at clinical visit (years)	0.01 (0.00, 0.02)	0.021
Education (1–4, lowest to highest)	−0.08 (−0.19, 0.03)	0.144
White vs. other racial and ethnic groups	−0.14 (−0.41, 0.13)	0.312
Diagnosis of endometriosis	0.17 (−0.09, 0.44)	0.207
Experience of childhood abuse	0.11 (−0.11, 0.34)	0.324

CI, confidence interval; PCS, Pain Catastrophizing Scale.

^a^ Missing values were imputed using multiple imputation with 10 imputation sets assuming multivariate normal distribution. All predicting variables, as well as auxiliary variables including major clinical presentation (pelvic pain vs. vulvar pain), experience of adult abuse (yes vs. no), ever use of tobacco (yes vs. no), BMI at the clinical visit (kg/m^2^), PHQ-2 screening score (ranging 0–6), GAD-2 screening score (ranging 0–6), clinical diagnosis of previous and/or current MDD, and clinical diagnosis of previous and/or GAD, were included in the imputation model for variables with missing value.

**Table 5:** Multiple linear regression model for the covariates-adjusted association between dysmenorrhea catastrophizing and chronic pelvic pain interference^a^.

Variables predicting chronic pelvic pain interference (0–10)	Beta (95% CI)	*P*
Dysmenorrhea catastrophizing (1–4, lowest to highest)	0.63 (0.06, 1.20)	0.032
Pain catastrophizing (PCS total score)	0.02 (−0.04, 0.07)	0.561
Pelvic pain intensity (0–4)	0.96 (0.28, 1.65)	0.006
Age at clinical visit (years)	−0.01 (−0.05, 0.03)	0.703
Education (1–4, lowest to highest)	−0.00 (−0.53, 0.53)	0.996
White vs. other racial and ethnic groups	0.13 (−1.34, 1.59)	0.864
Diagnosis of MDD	0.40 (−0.76, 1.56)	0.496
Diagnosis of GAD	−0.17 (−1.43, 1.09)	0.794
Experience of childhood abuse	0.98 (−0.25, 2.21)	0.117
Experience of adulthood abuse	0.34 (−0.86, 1.54)	0.579

CI, confidence interval; PCS, Pain Catastrophizing Scale; MDD, major depressive disorder; GAD, generalized anxiety disorder.

^a^ Missing values were imputed using multiple imputation with 10 imputation sets assuming multivariate normal distribution. All predicting variables, as well as auxiliary variables including diagnoses of bladder pain syndrome, irritable bowel syndrome, endometriosis, vulvodynia, myofascial pelvic pain, fibromyalgia, pelvic pain intensity measured by NRS, ever use of tobacco (yes vs. no), BMI at the clinical visit (kg/m^2^), PHQ-2 screening score (ranging 0–6), and GAD-2 screening score (ranging 0–6), were included in the imputation model for variables with missing value.

Table 4 presents results for the association between dysmenorrhea catastrophizing and dysmenorrhea interference. Adjusting for dysmenorrhea frequency, duration, and intensity, pain catastrophizing, age and education at the initial clinical visit, race and ethnicity, endometriosis diagnosis, and childhood abuse, each 1-score increase in dysmenorrhea catastrophizing was associated with 0.44 greater dysmenorrhea interference (95% CI = 0.29, 0.59, *P* < 0.001). In this fully adjusted model, dysmenorrhea catastrophizing was the most significant predictor of dysmenorrhea interference, followed by dysmenorrhea intensity. Pain catastrophizing was not predictive of dysmenorrhea interference. When removing dysmenorrhea catastrophizing from the model, pain catastrophizing was not predictive of dysmenorrhea interference either (data not shown).

Table 5 presents results for the association between dysmenorrhea catastrophizing and CPP-associated pain interference. Adjusting for pain catastrophizing, current pelvic pain intensity, age and education at the initial clinical visit, race and ethnicity, depression and anxiety, childhood abuse and adulthood abuse, each 1-score increase in dysmenorrhea catastrophizing was associated with 0.63 greater CPP-associated pain interference (95% CI = 0.06, 1.20, *P* = 0.032). Pain catastrophizing was not predictive of CPP-associated pain interference. When removing dysmenorrhea catastrophizing from the model, pain catastrophizing was not predictive of CPP-associated pain interference either (data not shown).

### 3.4. Exploratory analyses results

Table 6 shows results for our exploratory analyses examining predictors of dysmenorrhea catastrophizing. In simple linear regression models, other racial and ethnic groups (compared with non-Hispanic white), early onset of dysmenorrhea, more frequent dysmenorrhea, early starting of pain during a menstrual period, greater dysmenorrhea intensity, ever being prescribed opioid medication for dysmenorrhea management, and use of marijuana for dysmenorrhea management were associated with greater dysmenorrhea catastrophizing, with dysmenorrhea intensity explaining the largest variance of dysmenorrhea catastrophizing. In the multiple linear regression model, dysmenorrhea intensity remained the only significant predictor of dysmenorrhea catastrophizing.

**Table 6:** Predictors of dysmenorrhea catastrophizing, results from simple and multiple linear regression models^a^.

Predictors	Simple linear regression		Multiple linear regression^a^	
	Beta (95% CI)	*R* ^2^	Beta (95% CI)	*P*
Age (years)	−0.01 (−0.03, 0.00)	0.023		
Race and ethnicity: white vs. others	−0.68 (−1.18, −0.17)	0.065	−0.16 (−0.58, 0.26)	0.454
Education (1–4, lowest to highest)	−0.18 (−0.37, 0.01)	0.034		
Dysmenorrhea onset		0.082		0.430
With 1st/2nd period	1.15 (0.33, 1.96)		0.27 (−0.39, 0.93)	
≤1 years of 1st period	0.86 (−0.00, 1.71)		−0.00 (−0.70, 0.69)	
≤5 years of 1st period	0.71 (−0.31, 1.74)		0.17 (−0.61, 0.96)	
≥5 years of 1st period	Ref		Ref	
Dysmenorrhea frequency		0.310		0.911
Seldom/rarely	Ref		Ref	
During some periods	0.19 (−0.74, 1.12)		0.12 (−0.70, 0.94)	
During most periods	0.96 (0.11, 1.81)		0.22 (−0.55, 0.98)	
During every period	1.72 (0.97, 2.47)		0.27 (−0.50, 1.05)	
When dysmenorrhea usually started		0.068		0.933
The day the period started	Ref		Ref	
1–2 days before the period	0.31 (−0.14, 0.76)		−0.07 (−0.44, 0.30)	
≥3 days before the period	0.79 (0.21, 1.37)		−0.04 (−0.54, 0.46)	
Dysmenorrhea duration		0.307		0.080
1–2 days	Ref		Ref	
3–4 days	0.76 (0.35, 1.18)		0.23 (−0.17, 0.64)	
≥5 days	1.46 (1.02, 1.90)		0.57 (0.07, 1.08)	
Dysmenorrhea intensity		0.543		<0.001
Mild	Ref		Ref	
Moderate	0.48 (0.02, 0.95)		0.25 (−0.29, 0.79)	
Severe	1.79 (1.37, 2.20)		1.28 (0.70, 1.85)	
Worst pain imaginable	2.38 (1.64, 3.13)		1.62 (0.67, 2.56)	
Used prescribed opioids: yes vs. no	1.01 (0.31, 1.72)	0.074	0.22 (−0.41, 0.85)	0.482
Used marijuana: yes vs. no	0.96 (0.32, 1.60)	0.080	0.27 (−0.29, 0.84)	0.340
* *			*R-squared*	*0* *.* *63*

^a^ Models were performed in one complete dataset randomly selected from the 10 imputed datasets.

^b^ Only significant predictors of dysmenorrhea catastrophizing (*P* < 0.05) from the simple linear regression models entered the multiple linear regression model.

## 4. Discussion

To our knowledge, the current study is the first to explore the role of dysmenorrhea-specific catastrophizing in both dysmenorrhea and CPP associated pain interference, among a clinical sample of women with CPP. Dysmenorrhea catastrophizing was associated with both dysmenorrhea interference as well as CPP-associated pain interference. Dysmenorrhea intensity was identified as the most important predictor of dysmenorrhea catastrophizing, given its large explanatory power and significant association that persisted through multivariable analysis.

The cyclic nature of dysmenorrhea presents a unique challenge for younger females as they develop methods to cope with pain and distress; pain catastrophizing could be part of the coping framework. Women with higher catastrophizing have been shown to report greater menstrual pain and associated disability (25, 26). Among our clinical sample, dysmenorrhea catastrophizing was most predictive of dysmenorrhea interference, with a greater effect than dysmenorrhea intensity, highlighting the importance of targeting dysmenorrhea catastrophizing in mitigating the functional impact of dysmenorrhea. Since managing dysmenorrhea symptoms is an integrated part of managing CPP, interventions to reduce dysmenorrhea catastrophizing may be clinically important for improving patients' quality of life. A recent study reported that pain acceptance predicted better quality of life among women with primary dysmenorrhea (38). It is possible that improving pain acceptance among women with severe dysmenorrhea may help reduce dysmenorrhea catastrophizing. However, it should be noted that dysmenorrhea catastrophizing is highly influenced by dysmenorrhea severity, as shown in our exploratory analyses. One study reported that pain catastrophizing scores varied throughout the menstrual cycle, being highest on the first day of menstrual cycle and declining subsequently, especially for women with dysmenorrhea (39), suggesting potential influence of dysmenorrhea symptoms on pain catastrophizing. Our study highlights the importance of more effective management of dysmenorrhea symptoms, especially reducing dysmenorrhea intensity, for reducing dysmenorrhea interference.

We also found that less than half of our clinical sample sought treatment specifically for their dysmenorrhea symptoms, although the majority reported a history of severe dysmenorrhea. This may be due in part to the normalization of menstrual pain, which potentially results in missed opportunities for intervention. Of note, a small proportion of women reported being prescribed opioid medications for managing dysmenorrhea, despite the fact that opioids are not medically recommended for pelvic pain treatment. More than 10% of women reported using marijuana for managing dysmenorrhea. With the national trend in legalizing marijuana, its use among adolescents and young adults should be monitored and the role played by dysmenorrhea in the initiation of marijuana use may be recognized with future research.

Our study is the first to test whether dysmenorrhea-specific catastrophizing is associated with subsequent CPP-associated pain interference, complementing previous literature on pain catastrophizing and CPP (22, 40–48). One could argue that dysmenorrhea catastrophizing is simply reflecting pain catastrophizing, rather than being a new construct. However, dysmenorrhea catastrophizing and PCS scores were only moderately correlated with each other (*ρ* = 0.30) in our study, providing some evidence that these two did not fully overlap. More importantly, PCS scores were not predictive of either dysmenorrhea interference or CPP-associated pain interference in both models with and without the inclusion of dysmenorrhea catastrophizing, potentially suggesting that dysmenorrhea catastrophizing could be a more specific predictor of pelvic pain interference, compared with pain catastrophizing in general. It should be noted that we intended to measure dysmenorrhea-specific catastrophizing prior to the development of CPP, which turned out to be a salient predictor of CPP-associated pain interference, highlighting the need to target dysmenorrhea catastrophizing early on for ameliorating pelvic pain interference.

It is somewhat surprising that we did not observe a positive association between pain catastrophizing and CPP-associated pain interference, which could be due in part to the relatively small correlation between pain catastrophizing and pain interference (*ρ* = 0.26) in our sample. In a previous study, PCS scores were highly correlated with pain-related interference (*ρ* = 0.56) among women with endometriosis (46). Such difference could be due to random sampling or difference in the study sample (chronic pelvic/vulvar pain patients vs. women with endometriosis). It is also possible that pain catastrophizing explains part of CPP-associated pain interference. In addition to pain from gynecologic origin, CPP can originate from urogenital, gastrointestinal, and musculoskeletal systems, as well as external reproductive organs. The pain symptoms can be episodic, persistent, provoked, or situation-dependent (e.g., pain associated with sexual activities). The multifaceted pain generators and complex pain experiences of female CPP may result in a relatively smaller variance of CPP interference explained by pain catastrophizing.

Currently, the conceptual underpinnings of pain catastrophizing are still equivocal despite the extensively used measures such as the PCS (16). Pain catastrophizing has been conceptualized as a cognitive schemata (49), a coping strategy (50), a personality trait or situational state (51), a communal coping strategy (51), and most recently, as a broader concept incorporating emotional regulation, catastrophic worry, rumination, behavioral inhibition and behavioral activation, and interoceptive sensitivity (52). An important question is whether one conceptualization is more clinically useful which could potentially be indicated by its utility in predicting clinical outcomes and its ability to change. Context-specific catastrophizing may augment general pain catastrophizing in associating pain experiences and could be more amenable to change. Mathur et al. (2016) examined disease-related, non-disease-related, and situational catastrophizing in relation to pain in sickle cell disease (SCD) and found that SCD-specific catastrophizing was higher than general catastrophizing (i.e., PCS average score). The authors further suggested that context-specific anchors may be beneficial in predicting different aspects of the pain experience (53). Similarly, our findings suggest that among women with CPP it may be beneficial to assess dysmenorrhea-specific catastrophizing which corresponds better with pain symptoms and may have greater potential of modification.

Limitations of the current study should be considered when interpreting these findings. First, although we tried to elicit dysmenorrhea symptoms, catastrophizing, and interference prior to the development of non-cyclic CPP, participants' current CPP severity, pain catastrophizing, and dysmenorrhea symptom at the time of the clinical visit could influence their report of previous dysmenorrhea symptoms, catastrophizing, and interference. We also acknowledge that recalling dysmenorrhea experiences prior to developing chronic pelvic pain is very challenging. Longitudinal studies are needed to examine whether dysmenorrhea catastrophizing increases the risk of CPP incidence and severity among adolescent girls, and whether interventions to reduce dysmenorrhea catastrophizing prevent the development or reduce the severity of future CPP.

Second, we did not apply rigorous qualitative research methodology in item development for the dysmenorrhea catastrophizing and dysmenorrhea interference measures, and these developed measures have not been validated in other studies, although they were developed by a group of experts in gynecological care, clinical psychology, and population health, and have gone through multiple internal iterations. Among our clinical sample of women with CPP, dysmenorrhea catastrophizing and dysmenorrhea interference demonstrated excellent internal consistency and good evidence of construct validity (moderate to strong correlations with dysmenorrhea severity and treatment intensity). However, measurement validation studies are needed to further assess the content validity, reliability, and responsiveness of dysmenorrhea catastrophizing across both clinical and healthy samples. Future studies should also consider using the Dysmenorrhea Symptom Interference (DSI) Scale developed by Chen et al. (54) to better capture the comprehensive impact of dysmenorrhea on women's daily life.

Third, we were not able to precisely assess CPP specific interference as the BPI-PI is a general measure of pain interference and women with CPP are more likely to have overlapping chronic non-pelvic pain. The association observed between dysmenorrhea catastrophizing and CPP-associated pain interference in our study may reflect the effect of dysmenorrhea catastrophizing on chronic pain in our clinical population, which may have underestimated the effect of dysmenorrhea catastrophizing on CPP specific interference.

Fourth, the population in our study is very heterogeneous. Dysmenorrhea may have differential etiological associations with various types of CPP although dysmenorrhea-specific catastrophizing may have transdiagnostic influence on pain coping. Additionally, some women may experience amenorrhea due to the use of hormones to management their dysmenorrhea, which could be another source of heterogeneity that the current study was not able to account for. More research is required to determine whether amenorrhea modifies the association between dysmenorrhea catastrophizing and pelvic pain interference.

Additionally, although we based our hypothesis on the Fear-Avoidance Model of pain which posits that pain leads to pain catastrophizing, which in turn, leads to disability, it is possible that women who experience more pain interference are more likely to feel nervous about and overwhelmed by their menstrual pain. The cross-sectional design of our study is not able to rule out this reverse causal relationship.

Our findings have several implications for future research and clinical practice. The developed Dysmenorrhea Catastrophizing measure needs further validation among clinical, non-clinical, and younger populations. This measure was designed for retrospective use in the current study; future studies may adapt it for assessing ongoing dysmenorrhea and consider more intensive assessment timeframe. Longitudinal studies are needed to determine whether dysmenorrhea catastrophizing reinforces pain catastrophizing over time, and whether reducing dysmenorrhea catastrophizing could lower the incidence, severity, and disability of CPP. Our findings reveal that dysmenorrhea catastrophizing may be an important aspect that is related to pelvic pain disability. It is therefore important to incorporate dysmenorrhea specific catastrophizing into the comprehensive evaluation of dysmenorrhea. Interventions aimed at reducing dysmenorrhea catastrophizing and dysmenorrhea intensity may serve as effective strategies for reducing pelvic pain disability.

## 5. Conclusion

In this clinical sample of women presenting with chronic pelvic/vulvar pain in a tertiary gynecological pain clinic, dysmenorrhea catastrophizing was associated with greater dysmenorrhea interference as well as subsequent CPP-related interference. Dysmenorrhea intensity emerged as the most important predictor of dysmenorrhea catastrophizing. Interventions focused on reducing both dysmenorrhea symptom severity and dysmenorrhea catastrophizing are important to study with the goal to reduce pain interference associated with female pelvic pain across the lifespan.

## Data Availability

The raw data supporting the conclusions of this article will be made available by the authors, without undue reservation.
