# Supplementary material for: Dysmenorrhea catastrophizing and functional impairment in female pelvic pain
Source: Front Pain Res (Lausanne). 2023 Jan 6;3:1053026. doi: 10.3389/fpain.2022.1053026 (PMC9853896; doi:10.3389/fpain.2022.1053026)
Supplement: Supplementary file 1 [file Datasheet1.docx]

Supplementary Material

**Dysmenorrhea Questionnaire**

**Definitions:**

- **Period cramps** are crampy pains you get just before or during your period.
- **Chronic pelvic pain** is pain in the pelvis or vulva that is *not* related to your menstrual cycle, although you may still have period cramps.

1. Have you ever experienced period cramps?

- 1. No -------- Please skip all of the following questions.
  2. Yes

2. When did your period cramps begin?

- 1. With my first/second period
  2. Within one year after my first period
  3. Within five years after my first period
  4. Greater than five years after my first period

3. How old were you when you had your **first** menstrual period?

_____ years

4. How old were you when you **first** experienced period cramps?

_____years

5. How old were you when you began to experience the pelvic/vulvar pain **not** related to period cramps for which you have been referred to this practice?

_____years

The following questions ask about your **period cramps** BEFORE you developed the **chronic pelvic and/or vulvar pain** for which you have been referred to this practice. When answering these questions, please refer to your period experiences BEFORE your chronic pelvic and/or vulvar pain condition(s) began. For each question, please circle your answer choice.

6. **Prior to** developing your chronic pelvic and/or vulvar pain,

(1) How often did you experience period cramps?

- 1. During every period
  2. During most of my periods
  3. During some of my periods
  4. Seldom or rarely

(2) When did the period cramps usually start?

1. The day I got my period
2. 1-2 days before I got my period
3. 3 or more days before I got my period

(3) How long did the period cramps usually last?

1. 1 – 2 days
2. 3 – 4 days
3. 5 or more days

(4) How would you describe the overall intensity of your period cramps without medication?

1. Mild
2. Moderate
3. Severe
4. Worst pain imaginable

7. **Prior to** developing your chronic pelvic and/or vulvar pain, please rate how much the following statements applied to you **when you were experiencing the period cramps:**

|  | *Not at all* | *To a slight degree* | *To a moderate degree* | *To a great degree* |
| --- | --- | --- | --- | --- |
| a. It was awful, and I felt that it overwhelmed me | 1 | 2 | 3 | 4 |
| b. I was afraid that the pain would get worse | 1 | 2 | 3 | 4 |
| c. I kept thinking about how much it hurt | 1 | 2 | 3 | 4 |
| d. ***Before*** my period, I became nervous about my period cramps | 1 | 2 | 3 | 4 |

8. **Prior to** developing your chronic pelvic and/or vulvar pain, **when you were experiencing the period cramps**, how did the period cramps interfere with your:

|  | *Did not interfere at all* | *Interfered a little* | *Interfered a lot* | *Completely interfered* |
| --- | --- | --- | --- | --- |
| a. General daily activity (e.g., eating, bathing, dressing, walking) | 1 | 2 | 3 | 4 |
| b. School/work activity (e.g., attending classes, taking exams, performing normal work) | 1 | 2 | 3 | 4 |
| c. Relations with others (e.g., going out with friends, physical intimacy) | 1 | 2 | 3 | 4 |

9. **Prior to** developing your chronic pelvic and/or vulvar pain, did you ever seek medical treatment **specifically** for your period cramps?

1. Yes
2. No

10. **Prior to** developing your chronic pelvic and/or vulvar pain, have you ever used the following methods to **manage your period cramps** (please circle all that apply)?

- 1. Heating pad
  2. Over-the counter pain medications (e.g., Ibuprofen, Naproxen, Aspirin, Tylenol)
  3. Hormonal treatments (please check all that apply)
     - Birth control pills, patch, or ring
     - Progesterone only pills (e.g., Norethindrone/Aygestin, Provera, Megace)
     - Depo Provera (Depo shot)
     - Implant (e.g., Implanon, Nexplanon)
     - Progestin IUD (e.g., Mirena, Skyla, Liletta)
     - Lupron shot
  4. Prescription gabapentin, muscle relaxants, or NSAIDs
  5. Prescription opioid pain medications (e.g., Codeine, Hydrocodone, Oxycodone)
  6. Marijuana
  7. Transcutaneous electrical nerve stimulation (TENS)
  8. Surgical interventions (e.g., nerve ablation, spinal manipulation, laparoscopy)
  9. Complementary medicine (e.g., fish oil, herbs, acupuncture, yoga)

11. **Prior to** developing your chronic pelvic and/or vulvar pain, how have your period cramps changed over time since you first experienced them?

- 1. The cramps have worsened
  2. The cramps have stayed the same
  3. The cramps have gotten better
  4. The severity of cramps has fluctuated

**Supplementary Table 1.** Dysmenorrhea Catastrophizing and Dysmenorrhea Interference Scores according to Individual Strategies Used for Managing Dysmenorrhea

| **Strategies** | **Ever use** | **Dysmenorrhea catastrophizing** | **Dysmenorrhea interference** |
| --- | --- | --- | --- |
| Heating pad | Yes | 2.7 | 2.5 |
|  | No | 1.9 | 1.8 |
| Over-the counter pain medications | Yes | 2.6 | 2.4 |
|  | No | 1.4 | 1.4 |
| Birth control pills | Yes | 3.0 | 2.7 |
|  | No | 2.1 | 1.9 |
| Progesterone only pills | Yes | 3.2 | 2.8 |
|  | No | 2.5 | 2.3 |
| Depo Provera | Yes | 3.5 | 3.3 |
|  | No | 2.4 | 2.3 |
| Implant | Yes | 3.7 | 3.0 |
|  | No | 2.5 | 2.3 |
| Progestin IUD | Yes | 3.1 | 2.6 |
|  | No | 2.5 | 2.3 |
| Prescription gabapentin, muscle relaxants, or NSAIDs | Yes | 3.0 | 3.0 |
|  | No | 2.4 | 2.2 |
| Prescription opioid pain medications | Yes | 3.5 | 2.8 |
|  | No | 2.4 | 2.3 |
| Marijuana | Yes | 3.4 | 2.6 |
|  | No | 2.4 | 2.3 |
| Transcutaneous electrical nerve stimulation (TENS) | Yes | 3.6 | 3.3 |
|  | No | 2.5 | 2.3 |
| Surgical interventions | Yes | 3.3 | 2.8 |
|  | No | 2.4 | 2.3 |
| Complementary medicine | Yes | 3.1 | 2.7 |
|  | No | 2.4 | 2.3 |

**Supplementary Table 2.** Multiple Linear Regression Model for the Association between Dysmenorrhea Catastrophizing and Dysmenorrhea Interference, Excluding Women Experiencing Dysmenorrhea after 1 Year of First Menstrual Period (n = 87)^a^

| **Variables predicting dysmenorrhea interference (1–4)** | **Beta (95% CI)** | **P** |
| --- | --- | --- |
| Dysmenorrhea catastrophizing (1–4, lowest to highest) | 0.46 (0.29, 0.62) | < 0.001 |
| Dysmenorrhea frequency (1–4, lowest to highest) | 0.15 (-0.07, 0.37) | 0.184 |
| Dysmenorrhea duration (1–3, lowest to highest) | -0.19 (-0.40, 0.02) | 0.077 |
| Dysmenorrhea intensity (1–4, lowest to highest) | 0.22 (-0.01, 0.45) | 0.066 |
| Pain catastrophizing (PCS total score) | 0.01 (-0.01, 0.02) | 0.260 |
| Age at clinical visit (years) | 0.01 (0.00, 0.02) | 0.008 |
| Education (1–4, lowest to highest) | -0.09 (-0.22, 0.04) | 0.161 |
| White vs other racial and ethnic groups | -0.04 (-0.35, 0.27) | 0.782 |
| Diagnosis of endometriosis | 0.22 (-0.07, 0.52) | 0.137 |
| Experience of childhood abuse | 0.09 (-0.18, 0.36) | 0.508 |

CI = confidence interval; PCS = Pain Catastrophizing Scale.

^a^ Missing values were imputed using multiple imputation with 10 imputation sets assuming multivariate normal distribution. All predicting variables, as well as auxiliary variables including major clinical presentation (pelvic pain vs vulvar pain), experience of adult abuse (yes vs no), ever use of tobacco (yes vs no), BMI at the clinical visit (kg/m^2^), PHQ-2 screening score (ranging 0–6), GAD-2 screening score (ranging 0–6), clinical diagnosis of previous and/or current MDD, and clinical diagnosis of previous and/or GAD, were included in the imputation model for variables with missing value.

**Supplementary Table 3.** Multiple Linear Regression Model for the Association between Dysmenorrhea Catastrophizing and Chronic Pelvic Pain Interference, Excluding Women Experiencing Dysmenorrhea after 1 Year of First Menstrual Period (n = 87)^a^

| **Variables predicting chronic pelvic pain interference (0–10)** | **Beta (95% CI)** | **P** |
| --- | --- | --- |
| Dysmenorrhea catastrophizing (1–4, lowest to highest) | 0.65 (0.04, 1.26) | 0.037 |
| Pain catastrophizing (PCS total score) | 0.01 (-0.05, 0.07) | 0.704 |
| Pelvic pain intensity (0–4) | 1.14 (0.39, 1.89) | 0.003 |
| Age at clinical visit (years) | -0.02 (-0.06, 0.03) | 0.448 |
| Education (1–4, lowest to highest) | -0.07 (-0.65, 0.52) | 0.826 |
| White vs other racial and ethnic groups | 0.58 (-0.99, 2.14) | 0.470 |
| Diagnosis of MDD | 0.60 (-0.65, 1.85) | 0.350 |
| Diagnosis of GAD | 0.08 (-1.24, 1.41) | 0.902 |
| Experience of childhood abuse | 0.87 (-0.47, 2.20) | 0.204 |
| Experience of adulthood abuse | 0.38 (-0.90, 1.66) | 0.561 |

CI = confidence interval; PCS = Pain Catastrophizing Scale; MDD = major depressive disorder; GAD = generalized anxiety disorder.

^a^ Missing values were imputed using multiple imputation with 10 imputation sets assuming multivariate normal distribution. All predicting variables, as well as auxiliary variables including diagnoses of bladder pain syndrome, irritable bowel syndrome, endometriosis, vulvodynia, myofascial pelvic pain, fibromyalgia, pelvic pain intensity measured by NRS, ever use of tobacco (yes vs no), BMI at the clinical visit (kg/m^2^), PHQ-2 screening score (ranging 0–6), and GAD-2 screening score (ranging 0–6), were included in the imputation model for variables with missing value.

**Supplementary Table 4.** Multiple Linear Regression Model for the Association between Dysmenorrhea Catastrophizing and Dysmenorrhea Interference, Excluding Women who Rarely Experienced Dysmenorrhea (n = 97)^a^

| **Variables predicting dysmenorrhea interference (1–4)** | **Beta (95% CI)** | **P** |
| --- | --- | --- |
| Dysmenorrhea catastrophizing (1–4, lowest to highest) | 0.45 (0.29, 0.61) | < 0.001 |
| Dysmenorrhea frequency (1–3, lowest to highest) | 0.09 (-0.14, 0.31) | 0.466 |
| Dysmenorrhea duration (1–3, lowest to highest) | -0.16 (-0.35, 0.03) | 0.099 |
| Dysmenorrhea intensity (1–4, lowest to highest) | 0.27 (0.05, 0.49) | 0.016 |
| Pain catastrophizing (PCS total score) | 0.01 (-0.01, 0.02) | 0.345 |
| Age at clinical visit (years) | 0.01 (0.00, 0.02) | 0.007 |
| Education (1–4, lowest to highest) | -0.09 (-0.20, 0.03) | 0.127 |
| White vs other racial and ethnic groups | -0.12 (-0.40, 0.16) | 0.404 |
| Diagnosis of endometriosis | 0.22 (-0.05, 0.49) | 0.107 |
| Experience of childhood abuse | 0.15 (-0.09, 0.38) | 0.222 |

CI = confidence interval; PCS = Pain Catastrophizing Scale.

^a^ Missing values were imputed using multiple imputation with 10 imputation sets assuming multivariate normal distribution. All predicting variables, as well as auxiliary variables including major clinical presentation (pelvic pain vs vulvar pain), experience of adult abuse (yes vs no), ever use of tobacco (yes vs no), BMI at the clinical visit (kg/m^2^), PHQ-2 screening score (ranging 0–6), GAD-2 screening score (ranging 0–6), clinical diagnosis of previous and/or current MDD, and clinical diagnosis of previous and/or GAD, were included in the imputation model for variables with missing value.

**Supplementary Table 5.** Multiple Linear Regression Model for the Association between Dysmenorrhea Catastrophizing and Chronic Pelvic Pain Interference, Excluding Women who Rarely Experienced Dysmenorrhea (n = 97)^a^

| **Variables predicting chronic pelvic pain interference (0–10)** | **Beta (95% CI)** | **P** |
| --- | --- | --- |
| Dysmenorrhea catastrophizing (1–4, lowest to highest) | 0.61 (0.03, 1.20) | 0.040 |
| Pain catastrophizing (PCS total score) | 0.02 (-0.03, 0.07) | 0.420 |
| Pelvic pain intensity (0–4) | 1.02 (0.34, 1.70) | 0.003 |
| Age at clinical visit (years) | 0.01 (-0.03, 0.05) | 0.659 |
| Education (1–4, lowest to highest) | -0.14 (-0.69, 0.41) | 0.622 |
| White vs other racial and ethnic groups | 0.14 (-1.29, 1.58) | 0.844 |
| Diagnosis of MDD | 0.45 (-0.73, 1.62) | 0.455 |
| Diagnosis of GAD | -0.03 (-1.28, 1.22) | 0.960 |
| Experience of childhood abuse | 0.84 (-0.40, 2.09) | 0.185 |
| Experience of adulthood abuse | 0.26 (-0.95, 1.46) | 0.677 |

CI = confidence interval; PCS = Pain Catastrophizing Scale; MDD = major depressive disorder; GAD = generalized anxiety disorder.

^a^ Missing values were imputed using multiple imputation with 10 imputation sets assuming multivariate normal distribution. All predicting variables, as well as auxiliary variables including diagnoses of bladder pain syndrome, irritable bowel syndrome, endometriosis, vulvodynia, myofascial pelvic pain, fibromyalgia, pelvic pain intensity measured by NRS, ever use of tobacco (yes vs no), BMI at the clinical visit (kg/m^2^), PHQ-2 screening score (ranging 0–6), and GAD-2 screening score (ranging 0–6), were included in the imputation model for variables with missing value.
